# Reconstructing the biological invasion of noxious invasive weed *Parthenium hysterophorus* and invasion risk assessment in China

**DOI:** 10.3389/fpls.2024.1430576

**Published:** 2024-09-19

**Authors:** Huisen Zheng, Xinjie Mao, Yi Lin, Keyi Fu, Zanyi Qi, Yongbin Wu

**Affiliations:** College of Forestry and Landscape Architecture, South China Agricultural University, Guangzhou, China

**Keywords:** dispersal routes, climate change, MAXENT model, potential suitable area, Asteraceae

## Abstract

Invasive alien plants (IAPs) present a severe threat to native ecosystems and biodiversity. Comprehending the potential distribution patterns of these plant invaders and their responses to climate change is essential. *Parthenium hysterophorus*, native to the Americas, has become an aggressively invasive species since its introduction to China in the 1930s. This study aims to collect and reconstruct the historical occurrence and invasion of *P. hysterophorus*. Using the optimal MaxEnt model, the potential geographical distributions of *P. hysterophorus* were predicted based on screened species occurrences and environmental variables under the current and three future scenarios in the 2030s, 2050s, and 2070s (i.e., SSP1-2.6, SSP2-4.5, and SSP5-8.5), and the invasion risk of *P. hysterophorus* in Chinese cities, croplands, forests, and grasslands was assessed. The results show that: (1) The species initially invaded highly suitable areas and further spread to regions with non-analogous climate conditions. (2) Under the current climatic conditions, the overall potential distribution of *P. hysterophorus* is characterized by more in the southeast and less in the northwest. Climate variables, including mean annual temperature (bio1), precipitation in the wettest month (bio13), isothermality (bio3), and temperature seasonality (bio4), are the primary factors influencing its distribution. (3) The potential distribution of *P. hysterophorus* will expand further under future climate scenarios, particularly toward higher latitudes. (4) Forests and crop lands are the areas with the most serious potential invasion risk of *P. hysterophorus*. Therefore, we suggest that the government should strengthen the monitoring and management of *P. hysterophorus* to prevent its spread and protect agro-ecosystems and human habitats. Depending on the potential risk areas, measures such as quarantine, removal, and publicity should be taken to mitigate the threat of *P. hysterophorus* invasion and to raise awareness of *P. hysterophorus* invasion prevention.

## 1 Introduction

Biological invasion entails the intentional or unintentional introduction of an organism into an area conducive to its habitat and reproduction, leading to an expanding population and the gradual and steady expansion of its range (Pyšek et al., 2004). After extensive adaptation and dispersal, they pose a threat to the growth of local organisms and cause harm to the ecosystem (Costan et al., 2023; Daly et al., 2023; Derham et al., 2018). The rapid development of economic globalization has resulted in the emergence of biological invasion as a pervasive global issue. The number of invasive species is increasing, with a significant negative impact on the environment, economy and society in many countries and regions worldwide (Camacho-Cervantes and Wong, 2023; Cuthbert et al., 2022; Turbelin et al., 2023). The situation surrounding the prevention and management of invasive alien species is dire and complicated, and there is a concerns among governments and academics worldwide that this topic represents one of the major environmental challenges. Research suggests that biological invasions will become one of the foremost worldwide environmental challenges of the 21st century (Vantarová et al., 2023). It is estimated that the number of IAPs will continue to increase on most continents by 2050 (Sardain et al., 2019; Seebens et al., 2021). In China, IAPs of the Asteraceae family dominate among all invasive alien plant species, comprising 20.3% of the total (Weber et al., 2008; Zhang et al., 2021). Therefore, it is imperative that we synthesize the various factors and gain a comprehensive understanding of the changes in the distribution of IAPs under climate change to prevent the threat of further spread. Global warming and land use change are important drivers of the spread of IAPs (Brook et al., 2008; Corlett and Westcott, 2013; Humair et al., 2015). One of the reasons for the expansion of the area of suitable habitat for many species is the increase in temperature, which further affects the geographical distribution patterns of plants (Fernández et al., 2024; McDonald et al., 2023). Changes in land use led to the fragmentation of plant habitats, disrupting native ecosystems and creating opportunities for the invasion of alien plants (Thomas and Moloney, 2015). Drivers of changes in species distribution dynamics, influenced by future climate and land use changes, interact rather than act alone (Bowler et al., 2020). Therefore, conducting risk assessments of potential invasive areas under combined climate change and land use conditions can lay a stronger foundation for the future control and management of IAPs.

In recent years, predicting the dynamic potential distribution of IAPs can be done with different species distribution models (SDMs) (Baer and Gray, 2022; Mahmoodi et al., 2022; Yang et al., 2023). Using SDMs to estimate species’ range shifts and threat levels under future climate change scenarios is crucial for developing effective conservation and management decisions (Sofaer et al., 2019; Mahmoodi et al., 2023). Having the advantage of requiring presence-only data, simplicity in operation, and high accuracy, the MaxEnt model is widely employed (Rewicz et al., 2022). However, many studies often predict the potential suitable habitats for species under climate change scenarios without focusing on the practical aspects of land use for actual prevention and management efforts. This oversight could result in inconsistencies between predicted outcomes and actual application scenarios.

*Parthenium hysterophorus*, a member of the Asteraceae family, is a fast-growing, annual, poisonous, aggressive herb often found in wasteland, roadsides, riversides, and farmland. Currently, this invasive weed has spread to over 46 countries outside its native range (Mexico), particularly in Africa, Asia and Oceania (Mao et al., 2021). Its invasion has become a significant issue, affecting agricultural production, carrying capacity in rangelands, and human health across numerous Asian countries, especially Nepal, Bangladesh, and Pakistan. Owing to its invasiveness, spreading potential, economic, environmental, and health impacts, this invasive weed has been under focused monitoring in invasive regions, especially in China, where it has been placed on the ‘List of Priority Managed Invasive Alien Species’ for strict management and control (de Lange and van Wilgen, 2010; Shackleton and Shackleton, 2018; Wang et al., 2019). In the face of a deteriorating global environment, *P. hysterophorus* appears to be better adapted than native plants to survive rising temperatures and increased CO_2_ concentrations, which poses a threat to the growth of native plants, disrupts ecosystem functioning, and heightens the risk of reduced species diversity (Liu et al., 2017). It’s worth noting that changes in temperature and precipitation are vital climatic factors that determine plant distribution and invasiveness (Jaramillo and Cárdenas, 2013; Lamprecht et al., 2018; Vitasse et al., 2018).

This noxious invasive weed produces allelopathic effects chemicals that inhibit crop and pasture plants, potentially resulting in reduced crop yields and herbivore poisoning through accidental ingestion, hence impacting crop production and livestock development (Asif et al., 2017; Shabbir et al., 2019). Moreover, airborne pollen grains from this invasive weed may negatively affect humans, increasing the risk of severe atopic dermatitis or respiratory illnesses like hay fever and asthma in various individuals (Bajwa et al., 2016; Mao et al., 2021). This noxious invasive weed can contribute to the spread of malaria by providing more food and shelter for mosquitoes in East Africa (Nyasembe et al., 2015). *Parthenium hysterophorus* is a very harmful weed and has been included in the control list of China. However, its suitable and overlapping areas are still unknown. Therefore, it is crucial to study the species distribution of *P. hysterophorus* in China.

In this study, we used the optimal MaxEnt model to predict the potential geographic distribution pattern of *P. hysterophorus* in China under current and future climate change scenarios. The objectives of this study include: (1) mapping the current potential distribution and revealing important predictor variables of *P. hysterophorus*; (2) reconstructing the dispersal routes of *P. hysterophorus* in China; and (3) predicting changes in *P. hysterophorus*’s potential distribution under different future climate conditions; (4) assessing the risk of invasion by *P. hysterophorus* to cities, croplands, forests and grasslands in China. Our study is not only to assess the predicted changes in the potential distribution of *P. hysterophorus*, but also to identify the areas that should be monitored to prevent it from causing further negative impacts on the economy, ecology, and the safety of human life and health.

## 2 Materials and methods

### 2.1 Occurrence records of *P. hysterophorus*

**Figure 1:**
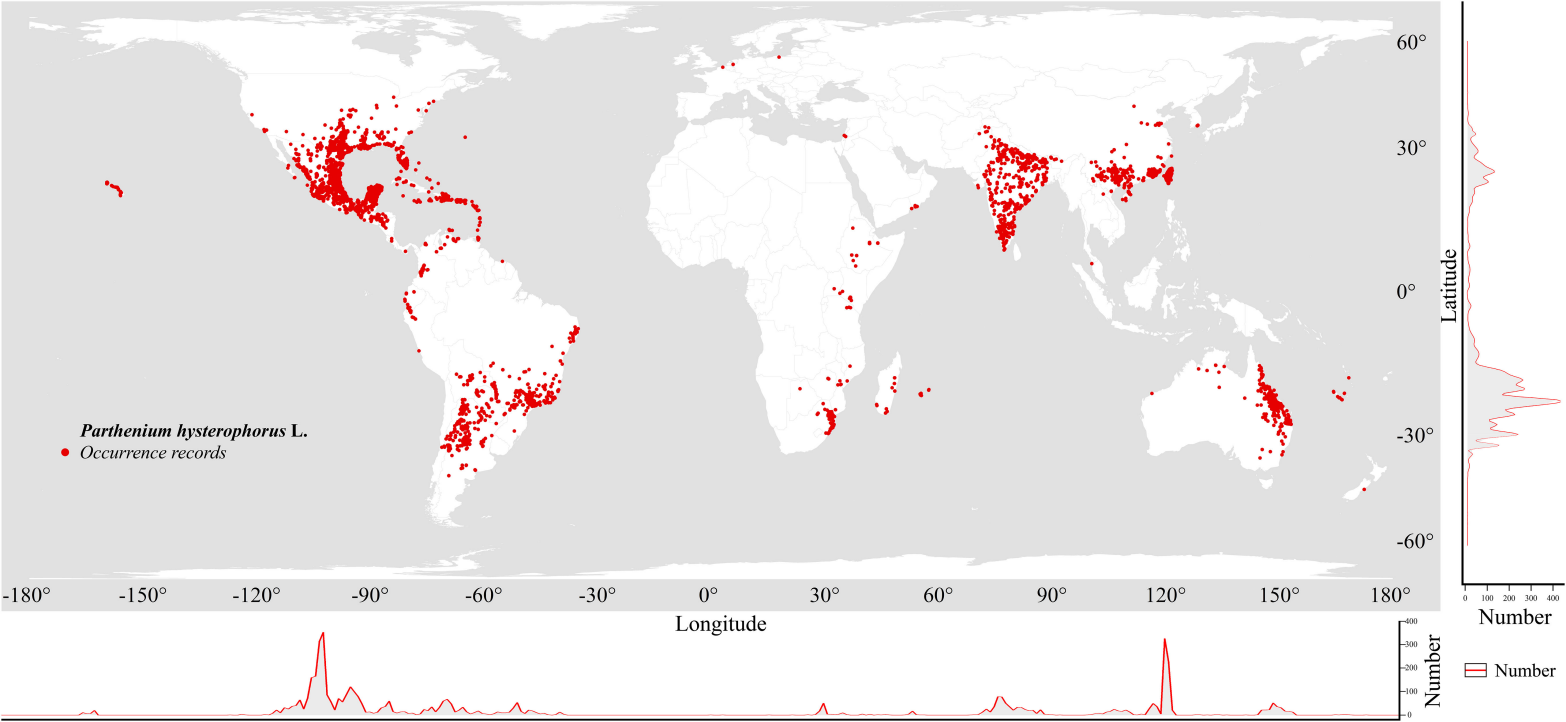
Screened geographical distribution of *P. hysterophorus* in the world.

The occurrence records of *P. hysterophorus* were obtained from the Global Biodiversity Information Facility (GBIF) (http://www.gbif.org/, accessed September 2023). Distribution data of *P. hysterophorus* from the China Virtual Herbarium (CVH) (http://www.cvh.ac.cn, accessed September 2023) were also utilized. Combining these with field surveys (**Supplementary Table S1**), all occurrence records were thoroughly examined, removing duplicate and non-terrestrial points, resulting in a preliminary dataset of 7533 occurrences. Spatial clustering of occurrences can lead to overfitting and inflated model performance values (Boria et al., 2014; Hijmans, 2012; Veloz, 2009). To avoid overfitting, we applied spatial autocorrelation reduction techniques (Boria et al., 2014; Phillips et al., 2009). The records were spatially filtered using ArcGIS 10.8, with SDMtoolbox v2.6 (Brown et al., 2017) employed to retain only one species distribution point within every 2.5 arc-minutes, resulting in the final dataset of 4,822 distribution points for *P. hysterophorus*. Finally, the final dataset was saved for subsequent MaxEnt model construction (**Figure 1**).

### 2.2 Dispersal routes of *P. hysterophorus*

*Parthenium hysterophorus* occurrence records were collected from CNKI, CVH, and official reports. The dates of the earliest published articles and the uploaded specimens or images can be employed as evidence to confirm the time of the invasion of *P. hysterophorus* in China. The dispersal routes for China were reconstructed using the minimum cost arborescence approach, as implemented in the ‘ecospat v4.1’ package (Hordijk and Broennimann, 2012; Marchioro and Krechemer, 2024). Occurrence times were randomly reassigned to available locations, with a subsequent comparison of the minimum and observed value of the total length of the dispersal route. Irrespective of the actual time of occurrence, the nearest neighboring site can be chosen as the antecedent to achieve the minimum possible value. The randomization process was conducted 100 times, culminating in the creation of a histogram that illustrates the obtained total dispersal routes for each iteration.

### 2.3 Environmental variables

This study considered four environmental factors highly correlated with the life cycle and geographic distribution of *P. hysterophorus*, and modeling selected 27 different variables (**Table 1**). These include 19 bioclimatic variables (bio1-bio19), 3 terrain variables (dem, slope, aspect), 4 soil variables (sand, silt, clay, pH), and NDVI (**Table 1**). Natural environmental factors can be divided into four categories, climate factors, soil factors, topographic factors, and NDVI. Climate factors are the most important drivers affecting IAPs dispersal, and soil, terrain, and NDVI also play an important role for them dispersal (Khan et al., 2019; Shiferaw et al., 2019). The 19 bioclimatic variables were downloaded from WorldClim 2.1 (https://www.worldclim.org/), while the 3 terrain variables, including dem (https://cmr.earthdata.nasa.gov/search/concepts/C1546314043-LPDAAC_ECS.html), slope, and aspect, 4 soil variables, including sand, silt, clay, and pH (https://www.isric.org/), and an NDVI (https://data.nasa.gov/dataset/MODIS-Terra-Vegetation-Indices-16-Day-L3-Global-50/diay-vffa/data) were obtained from Google Earth Engine processing (**Supplementary Table S2**).

**Table 1:** The predictor variables used in this study.

Predictor variables	Abbreviations	Final inclusion
Annual Mean Temperature (°C)	bio1	Yes
Mean Diurnal Range (°C)	bio2	
Isothermality (bio2/bio7) × 100	bio3	Yes
Temperature Seasonality (SD × 100)	bio4	Yes
Max Temperature of Warmest Month (°C)	bio5	
Min Temperature of Coldest Month (°C)	bio6	
Temperature Annual Range (bio5-bio6) (°C)	bio7	
Mean Temperature of Wettest Quarter (°C)	bio8	Yes
Mean Temperature of Driest Quarter (°C)	bio9	
Mean Temperature of Warmest Quarter (°C)	bio10	
Mean Temperature of Coldest Quarter (°C)	bio11	
Annual Precipitation (mm)	bio12	
Precipitation of Wettest Month (mm)	bio13	Yes
Precipitation of Driest Month (mm)	bio14	
Precipitation Seasonality (Coefficient of Variation)	bio15	
Precipitation of Wettest Quarter (mm)	bio16	
Precipitation of Driest Quarter (mm)	bio17	
Precipitation of Warmest Quarter (mm)	bio18	
Precipitation of Coldest Quarter (mm)	bio19	Yes
Digital Elevation Model	dem	
Slope	slope	
Aspect	aspect	
Normalized Difference Vegetation Index	NDVI	Yes
Proportion of sand particles (>0.05mm) in the fine earth fraction	sand	Yes
Proportion of clay particles (<0.002mm) in the fine earth fraction	clay	
Proportion of silt particles (0.002-0.05mm) in the fine earth fraction	silt	
Soil pH	pH	

The current (1970-2000) climate data includes 19 bioclimatic variables. The future climate data come from the Beijing Climate Center Climate System Model 2 Medium Resolution (BCC-CSM2-MR), which is a medium-resolution climate system model of the National Climate Center in the Sixth International Coupled Model Intercomparison Project (CMIP6), for three time periods: 2021-2040, 2041-2060, and 2061-2080, respectively. BCC-CSM2-MR has seen widespread application in research across East Asia and has demonstrated strong performance in China (Qiu et al., 2023; Wu et al., 2019, 2021). The future climate data of CMIP6 contains three shared socioeconomic pathways (SSPs), namely SSP1-2.6, SSP2-4.5, and SSP5-8.5, representing optimistic, moderate, and pessimistic scenarios, respectively (Eyring et al., 2016). To avoid multicollinearity among environmental factors causing model overfitting, this study, all species occurrences of *P. hysterophorus* and related environmental factors were entered into MaxEnt 3.4.3 to establish initial Species Distribution Models (SDMs) were used to determine the percent contributions of each environmental factor (**Supplementary Table S3**). Additionally, Pearson correlation analysis on the environmental variables was conducted using the ‘ggcorrplot v0.1.4.1’ package (Wickham, 2016) (**Supplementary Figure S1**). Environmental factors with a correlation coefficient |r| ≥ 0.8 and low contribution to the model were excluded from the modeling process (Brun et al., 2020). As a result, a set of 8 environmentally significant variables, both statistically and biologically relevant, was chosen for the model (**Table 1**). These selected variables had a spatial resolution of 2.5 arc minutes spatial resolution, ensuring that the model’s predictions were based on meaningful and non-redundant environmental data.

### 2.4 Modeling approach

The SDMs were developed with the maximum entropy algorithm implemented in software MaxEnt v.3.4.3 (Phillips et al., 2006b). The ‘ENMeval v2.0’ package (Kass et al., 2021) was used to calculate the regularization multipliers (RMs) and feature combinations (FCs) in the MaxEnt model (Warren et al., 2010), which can optimize the model and prevent overfitting, ensuring that the model’s predictions are accurate. By adjusting the RMs and FCs of the MaxEnt model, it becomes more reliable in predicting the potential distribution of *P. hysterophorus* under varying environmental conditions and climate scenarios. To account for spatial autocorrelation, the ‘checkerboard2’ blocking method (k = 4) was used for model training and validation (Fielding and Bell, 1997; Hastie et al., 2009; Peterson et al., 2011). The features of the MaxEnt model include linear (L), quadratic (Q), hinge (H), product (P), and threshold (T). This study tested 9 different feature combinations (L, LH, LQ, LQH, LQHP, LQHPT, LQP, QHP, QHPT) and 8 regularization multipliers ranging from 0.5 to 4 with a 0.5 interval (Maruthadurai et al., 2023). Subsequently, the ‘ENMeval’ package tested the above 72 combinations for parameter tuning, ultimately selecting the best parameter combination with ΔAICc=0 for MaxEnt modeling. Among them, ΔAICc is a measure of the goodness of fit of statistical models, which can weigh the complexity of the model and the goodness of the fitted data, and it prioritizes the model with the smallest ΔAICc value (i.e., deltaAICc=0) (Akaike, 1998). When the feature combination is QHP and the regularization multiplier is 0.5, ΔAICc=0.

MaxEnt was configured to import distribution and environmental data, with 25% of the distribution data selected for model testing and 75% for training. The model was trained for 10 repetitions (Phillips et al., 2006b), generating response curves and conducting jack-knife analysis to measure the importance of environmental variables (Elith et al., 2011).

### 2.5 Modeling evaluation and analysis

The accuracy of the model results was assessed using the Area Under the Curve (AUC) in the Receiver Operating Characteristic (ROC) curve analysis generated by MaxEnt (Phillips et al., 2006b). A higher AUC value indicates better prediction performance, with values below 0.6 indicating a failed prediction (Peterson et al., 2008). Additionally, the “ecospat” package was used to calculate the True Skill Statistic (TSS) and Kappa to assess the accuracy of the MaxEnt model (Allouche et al., 2006).

The result from the MaxEnt model was imported into ArcGIS and processed using Jenk’s natural breaks to reclass. These results were divided into four classes: areas with no suitable (0-0.11), poorly suitable (0.11-0.28), moderately suitable (0.28-0.50), and highly suitable (0.5-1). The area of different suitable was calculated by the raster calculator.

Species suitable(presence/absence) were classified using binary suitability maps: regions with a potential distribution probability <0.11 were set as absence and assigned a value of 0; regions with a potential distribution probability ≥0.11 were set as presence and assigned a value of 1, to obtain the binary absence/presence map matrix for each period. The results under current climate conditions with those under different future climate scenarios were calculated using ArcGIS.

Use the “Centroid Changes” tool in SDMtoolbox to calculate the change in potential distribution between two binary SDMs. This analysis can illuminate a trend in the potential distribution of *P. hysterophorus* observed in the period and reveal the impact of environmental changes on its potential distribution over time.

### 2.6 Persistent suitable areas identification

For *P. hysterophorus*, the persistent suitable areas were analyzed by overlaying their current potential suitable areas with future suitable areas in ArcGIS 10.8, with the overlapping areas identified as persistent suitable areas. The future suitable areas here refer to the overlap areas of the three emission scenarios (SSP1-2.6, SSP2-4.5, and SSP5-8.5) for the three decades (2030s, 2050s, and 2070s).

### 2.7 Invasion risk to cities, croplands, forests, and grasslands

*Parthenium hysterophorus* grows mainly in the wild on wasteland and roads and spreads to cities, croplands, forests, and grasslands through natural pathways and human activities. Given its hazardous nature, we evaluated the potential invasion risk of *P. hysterophorus* in these specific areas across China. We processed binary suitability and land-use distribution maps (https://zenodo.org/records/8176941) at a uniform spatial resolution, with overlay analysis applied to both datasets to ascertain the potential distribution of each land category exposed to suitable habitats for *P. hysterophorus* and calculate the total area of each land type exposed to the extent of the invasion.

## 3 Result

### 3.1 Model accuracy evaluation

The results reveal that the optimized MaxEnt model achieves a mean AUC of 0.838, with maximum Kappa and TSS values of 0.736 and 0.787, respectively. These metrics indicate that the model possesses high predictive accuracy and is reliably precise in its predictions.

### 3.2 Dispersal routes

**Figure 2:**
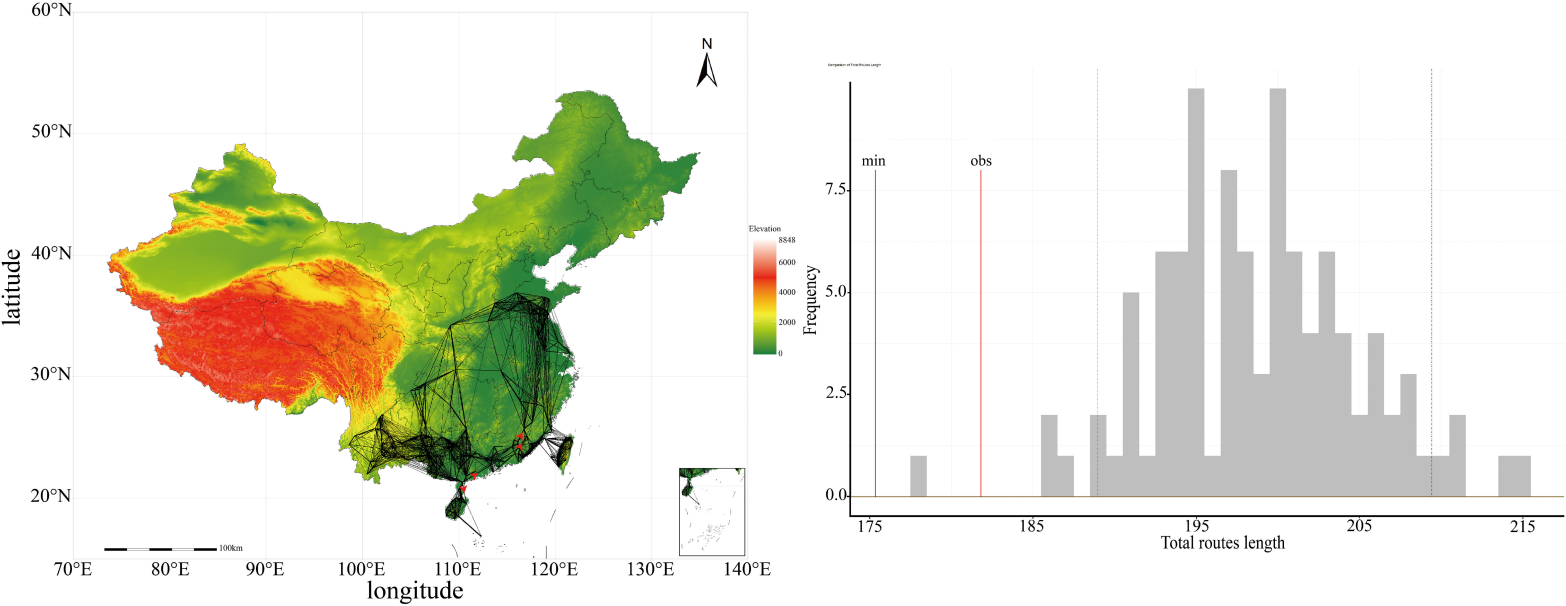
The dispersal pattern of *P. hysterophorus* during the biological invasion in China.

In 1926, the first specimen of *P. hysterophorus* was documented in China’s Yunnan Province, subsequently being identified in most provinces. Taiwan Province has been the most affected by the invasion, with *P. hysterophorus* expanding throughout the island since 1988. Subsequently, the provinces of Guangxi, Fujian, Guangdong, Yunnan, Hainan, and Guizhou have also become crucial areas for the invasion of *P. hysterophorus*. The reconstruction of the dispersal routes of *P. hysterophorus* is shown in the **Figure 2**. Our analysis reveals that the observed dispersal route lengths are relatively consistent with the simulated stochastic dispersal process, with the observed values (red line) positioned close to the simulated data (grey histogram), suggests that there is structured pattern in the dispersal dynamics of *P. hysterophorus* in China. A significant divergence between the minimum dispersal route length (blue line) and the observed dispersal route length (red line) suggests that *P. hysterophorus* spread over long distances in the species’ overall dispersal pattern.

### 3.3 Current predicted potential range

**Figure 3:**
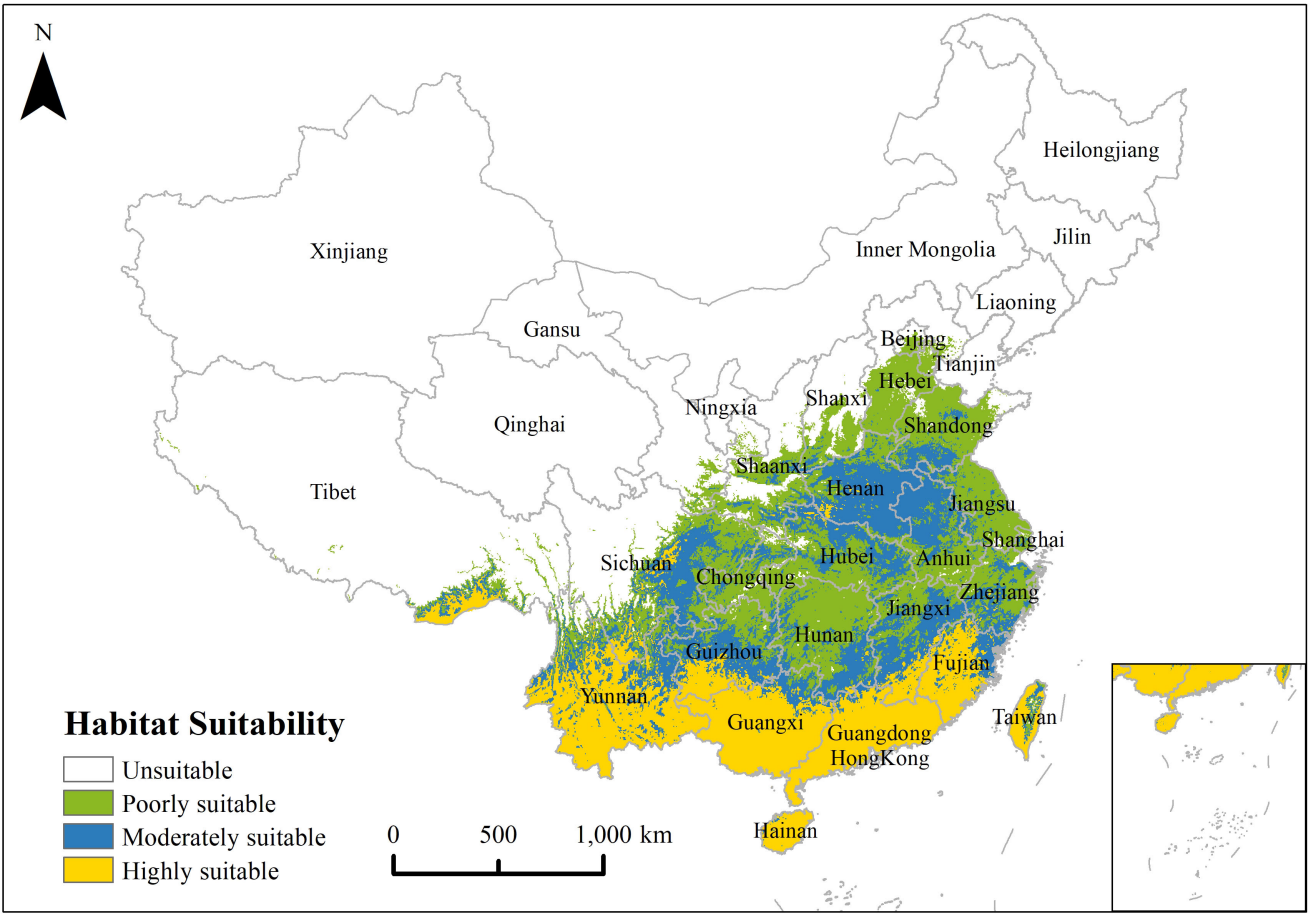
Potential distribution pattern of *P. hysterophorus* under current climate.

Under the environmental factors and current climate, the MaxEnt model results indicated that the overall potential distribution characteristics of *P. hysterophorus* were more in southeast and less in northwest (**Figure 3**). The suitable growth intensity and suitable growth area gradually decreased from southeast to northwest, and the areas with highly suitable were primarily located in Hainan, Guangdong, Guangxi, Yunnan, Fujian, Taiwan, south Guizhou, southeast Tibet and east Sichuan. Areas with poorly and moderately suitable conditions are mainly concentrated in Zhejiang, Jiangxi, Anhui, Shanghai, Jiangsu, Shandong, Henan, Hubei, Hunan, Chongqing, Beijing, Tianjin, Shaanxi and the southern part of Shanxi. According to the statistical results of ArcGIS raster calculator, the poorly, moderately and highly suitable habitats are 78.20×10^4^ km^2^, 83.37×10^4^ km^2^ and 116.63×10^4^ km^2^, respectively, accounting for 8.12%, 8.66% and 12.12% of China’s total area.

### 3.4 Significant environmental variables

**Figure 4:**
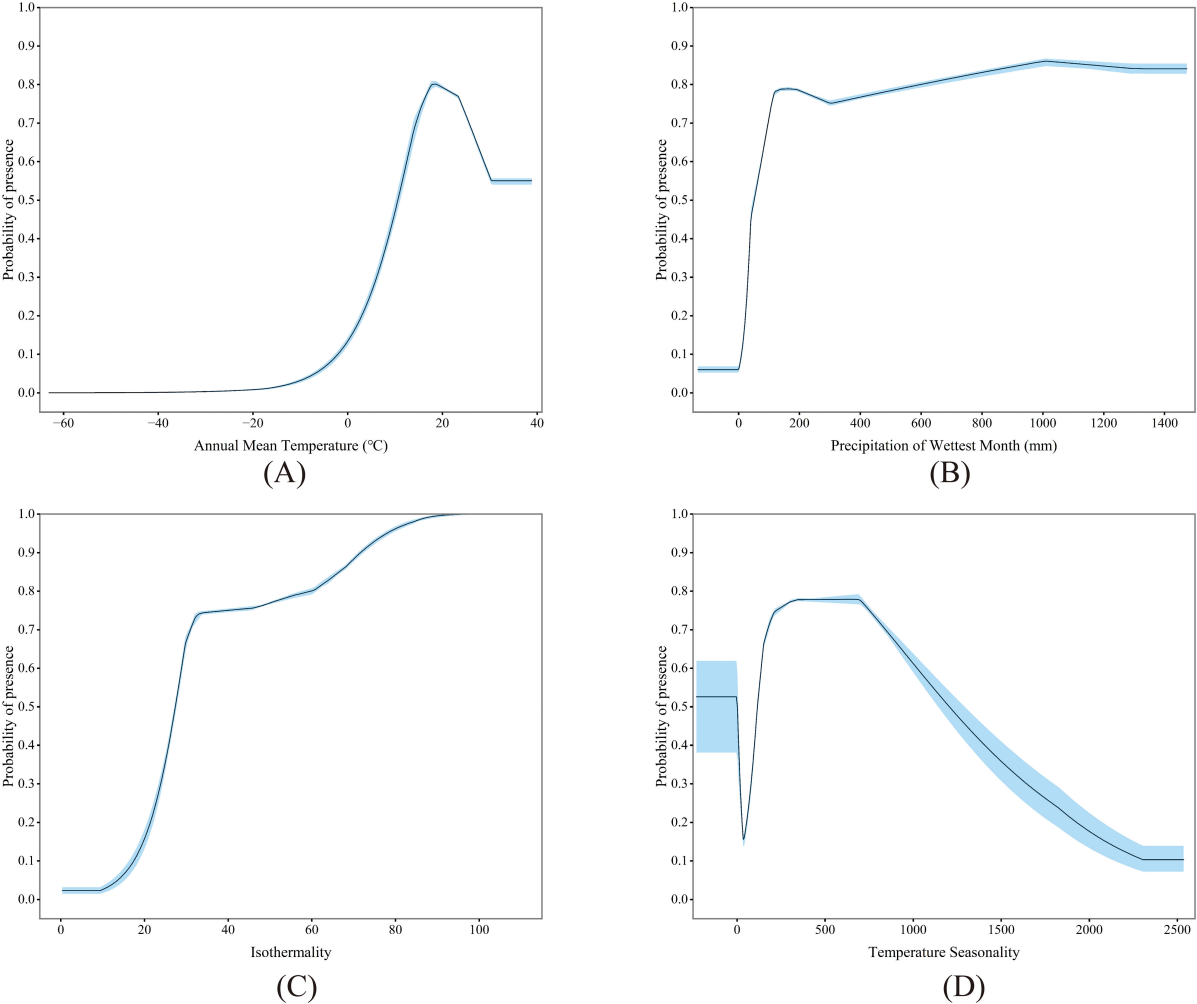
Significant environmental variables, including bio1 **(A)**, bio13 **(B)**, bio3 **(C)** and bio4 **(D)**.

The MaxEnt model’s variable contribution analysis highlights climate as the pivotal environmental factor influencing the potential distribution for *P. hysterophorus* in China (**Figure 4**). Significantly, the annual mean temperature (bio1) and precipitation in the wettest month (bio13) emerge as the dominant climate variables, collectively accounting for 71% of the model’s contribution. These factors stand as the most crucial predictors for the potential distribution of *P. hysterophorus*. Additionally, isothermality (bio3) and temperature seasonality (bio4) contribute 17.7% to the model, underscoring the importance of temperature fluctuations in affecting its potential distribution. Besides, NDVI and soil sand content are also among the factors affecting the potential distribution of *P. hysterophorus*.

The response curves between environmental variables and the presence of *P. hysterophorus* in this study are shown. For annual mean temperature (bio1), the high suitability zone (>0.5) ranged from 10.69 to 38.81°C, with the peak suitability at 18.44°C (**Figure 4A**). For precipitation in the wettest month (bio13), the high suitability zone extended from 10.49 to 1472.90 mm, with the maximum suitability reached at 1010 mm (**Figure 4B**). For isothermality (bio3) and temperature seasonality (bio4), the high suitability zones were identified with ranges of 27.32 to 109.06 and -230.48 to 1.84, as well as 118.00 to 1202.16, with peak suitability noted at 109.06 and 665.61, respectively (**Figures 4C, D**).

### 3.5 Potential distribution of *P. hysterophorus* under future climate scenarios

**Figure 5:**
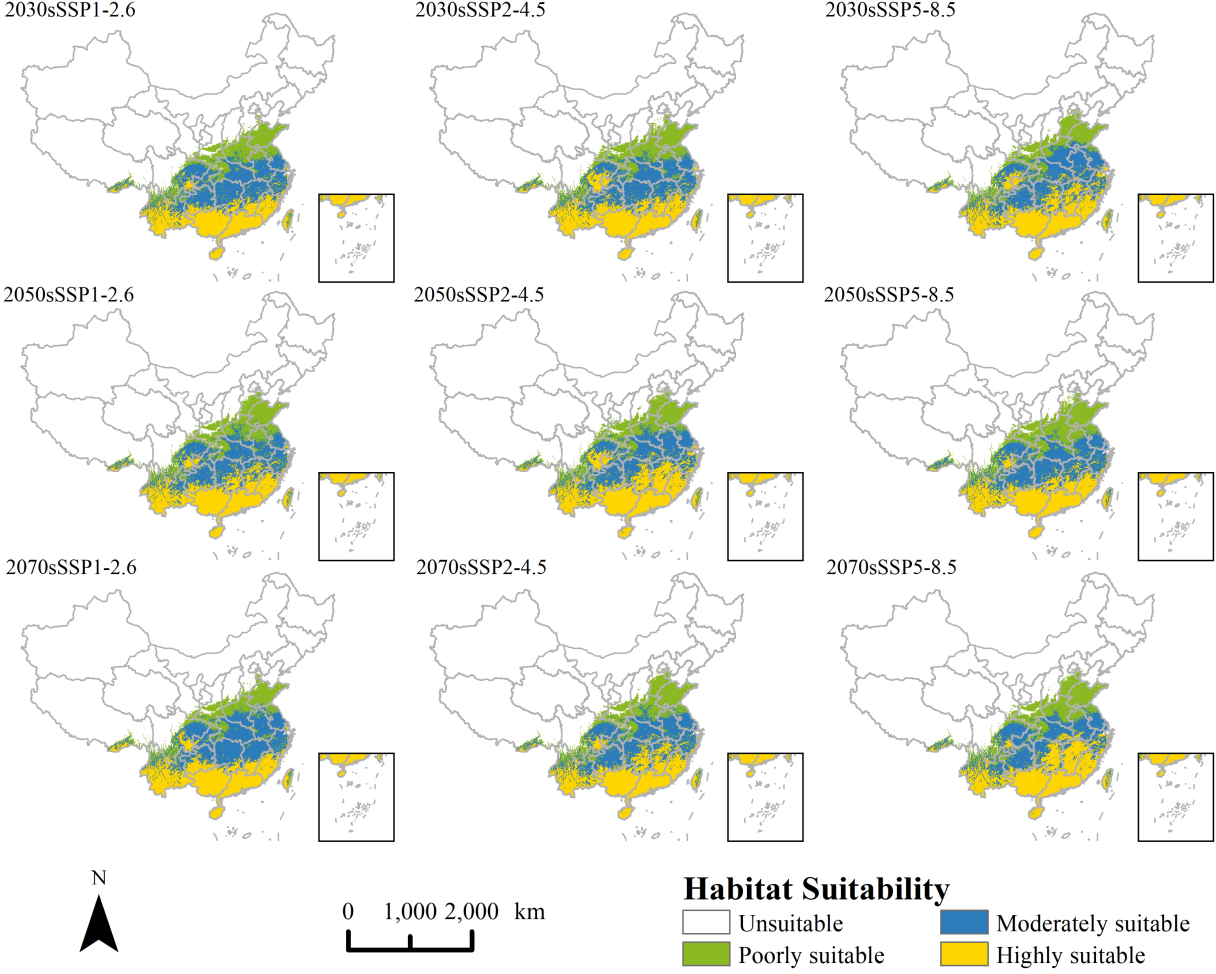
Potential distribution of *P. hysterophorus* under nine different future climatic conditions.

**Figure 6:**
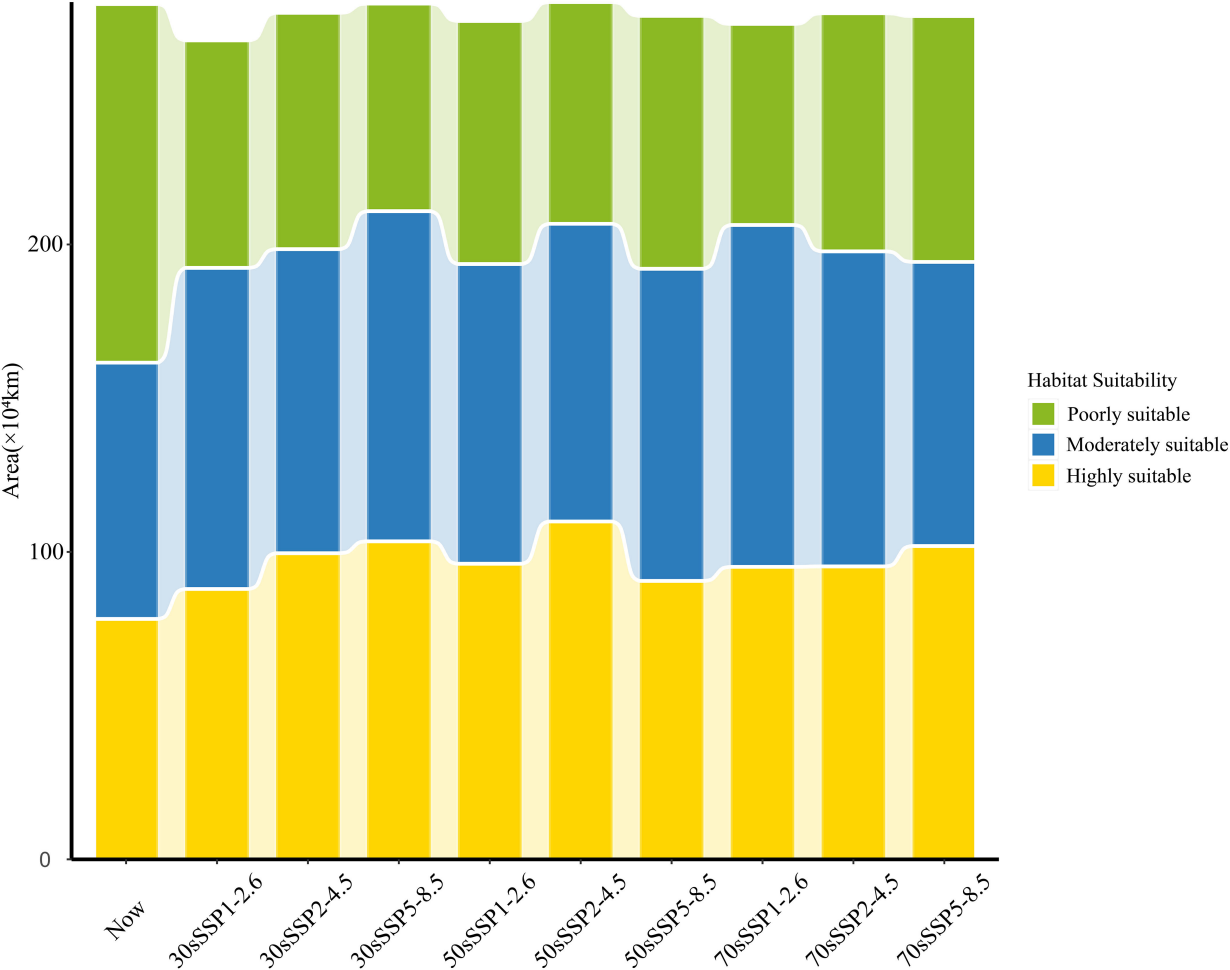
The distribution areas of *P. hysterophorus* under nine different future climatic conditions.

Under future climate scenarios, the projected invasion area of *P. hysterophorus* remains similar to the current potential distribution, with a concentration in the southeast and a decrease in the northwest (**Figure 5**). The total potential suitable area changes between -4.2% and 0.2%, indicating a relatively minor contraction in the projected area of *P. hysterophorus* (**Figure 6**). However, the comparison of different levels of habitable area shows that the poorly suitable habitat areas exhibited a downward trend over time, and the range is shrinking. The moderately and highly suitable habitats showed an increasing trend, and the range gradually expanded from low latitude to middle and high latitude.

There was an anticipated general increase in moderately and highly suitable areas by 2030 across all SSPs scenarios for *P. hysterophorus*, with a corresponding decrease in poorly suitable areas. Notably, the Sichuan Basin sees a significant increase in suitability, while Henan Province experiences a decrease. Under SSP5-8.5, the moderately and highly suitable areas peak at 107×10^4^ km^2^ and 103×10^4^ km², respectively, comprising 11.16% and 10.75% of China’s total area. This represents increases of 2.49% and 2.62% compared to the current climate.

Under the SSP1-2.6, SSP2-4.5, and SSP5-8.5 scenarios, by 2050, the overall change of the invaded area of *P. hysterophorus* exhibited a trend of descending after ascending. Under the SSP2-4.5 and SSP5-8.5, the moderately and highly suitable areas of *P. hysterophorus* reached the maximum value, which was 102×10^4^ and 110×10^4^ km^2^, respectively, accounting for 10.55% and 11.42% of the total area of China, and the moderately and highly suitable were 1.89% and 3.29% more, respectively, when compared with the current climate.

By 2070, the trend shows an increase in both highly and poorly suitable areas, which may indicate a divergence in habitat suitability, with some regions becoming more conducive to *P. hysterophorus* growth and others less so. The expansion of highly suitable areas is mainly in Jiangxi and Hunan provinces, with poorly suitable areas growing in Henan. Under SSP1-2.6 and SSP5-8.5, the poorly and highly suitable areas reach their maximum at 111×10^4^ km² and 102×10^4^ km², respectively, representing 11.56% and 10.59% of China’s total area. These are increases of 2.89% and 2.46% over the current climate.

**Figure 7:**
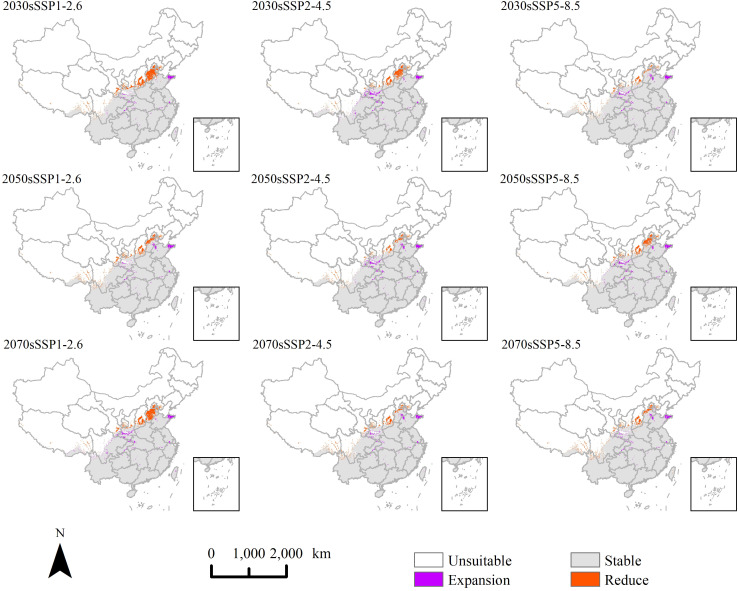
The change of potential suitable area of *P. hysterophorus* under different climate change scenarios in China.

In order to identify changes in suitable areas for *P. hysterophorus* under the current climate under future climate scenarios, we mapped areas that are unsuitable (“reduce” areas), suitable (“expansion” areas), and areas that remain stable (**Figure 7**). The findings indicate that the spatial distribution of potential suitable habitats varies minimally under different climate change scenarios. This trend mirrors the current expansion of suitable habitat northward. Newly suitable regions predominantly encompass Shandong, Hebei, Shaanxi, Hubei, and Chengdu. The newly unsuitable areas are primarily located in Hebei, Shanxi, Gansu, and Tibet.

**Figure 8:**
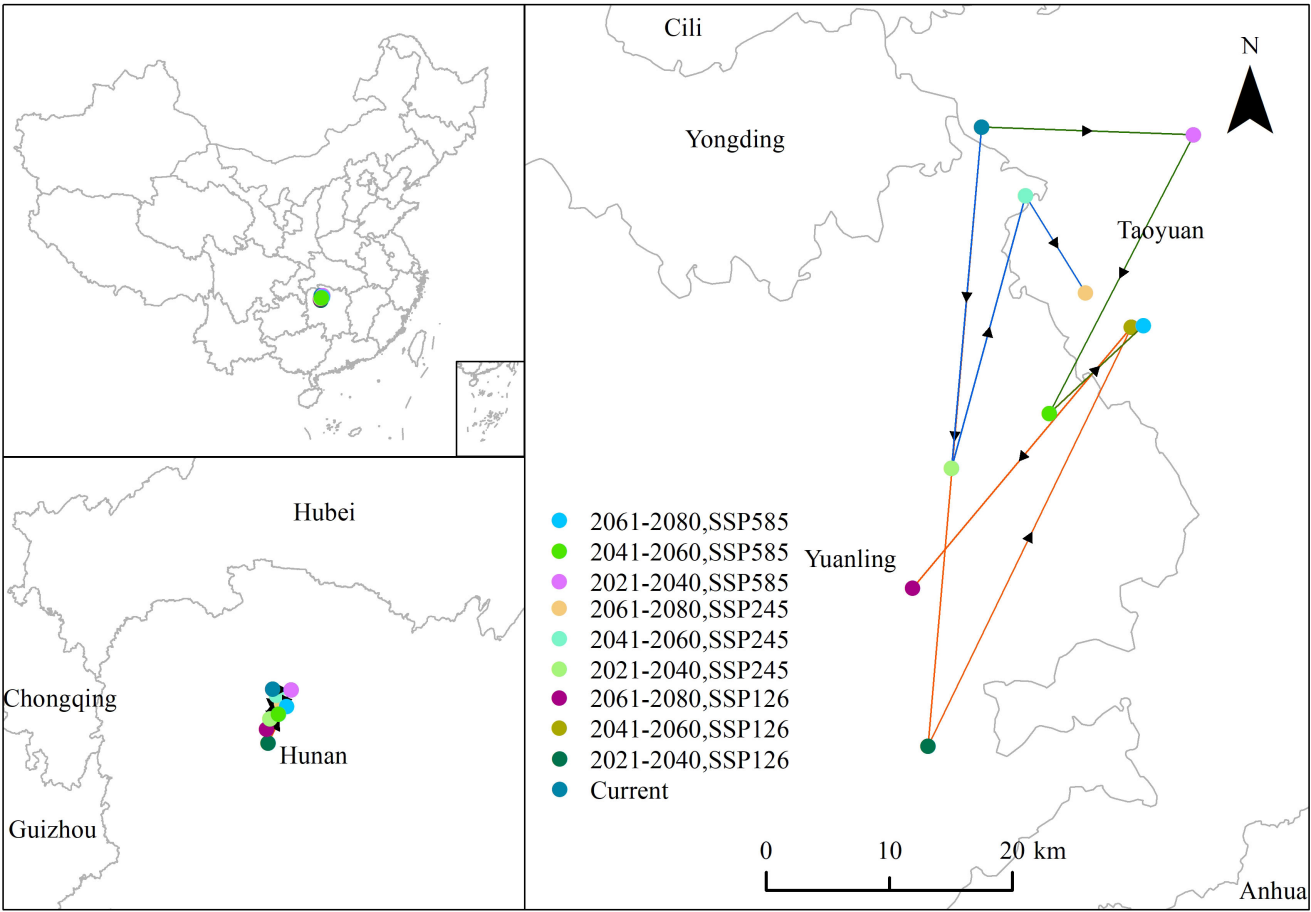
The distribution centers of *P. hysterophorus* under current nine future climatic conditions.

According to **Figure 8**, climate change has driven the potential distribution center of *P. hysterophorus* southward. The current center is in Taoyuan County, Changde City, Hunan Province, at coordinates 29°1’ 53.43”N, 110°55’ 40.13”E. Under the SSP1-2.6 scenario, the center moved 50.48 km southwest to Yuanling County, Huaihua City (28°34’ 38.99” N, 110°53’ 1.03”E), then 37.85 km northeast back to Taoyuan County (28°53’ 5.26” N, 111°3’ 10.58”E), and finally 27.67 km southwest again to Yuanling County (28°41’ 35.82” N, 110°52’ 15.22” E). Under the SSP2-4.5 pathway, the center shifted 27.82 km southwest to Yuanling County (28°46’ 52.99” N, 110°54’ 10.67”E), then 22.91 km northeast within the same county (28°58’ 51.59” N, 110°57’ 52.14”E), and 9.27 km southeast towards Taoyuan County (28°54’ 35.61” N, 111°0’ 52.50” E). Under SSP5-8.5, the center moved 17.26 km northwest to Taoyuan County (29°1’ 32.99”N, 111°6’ 17.89”E), then 25.50 km southwest to Yuanling County (28°49’ 17.21” N, 110°59’ 4.56”E), and 10.46 km northeast back to Taoyuan County (28°53’ 9.30” N, 111°3’ 46.57”E). The most considerable shift, approximately 50.48 km, occurred under SSP1-2.6 in the 2030s. In summary, the potential distribution center of *P. hysterophorus* has generally moved southward, with the main potential distribution area expanding due to climate change.

### 3.6 Risk of invasion of cities, croplands, forests and grasslands by *P. hysterophorus*

**Figure 9:**
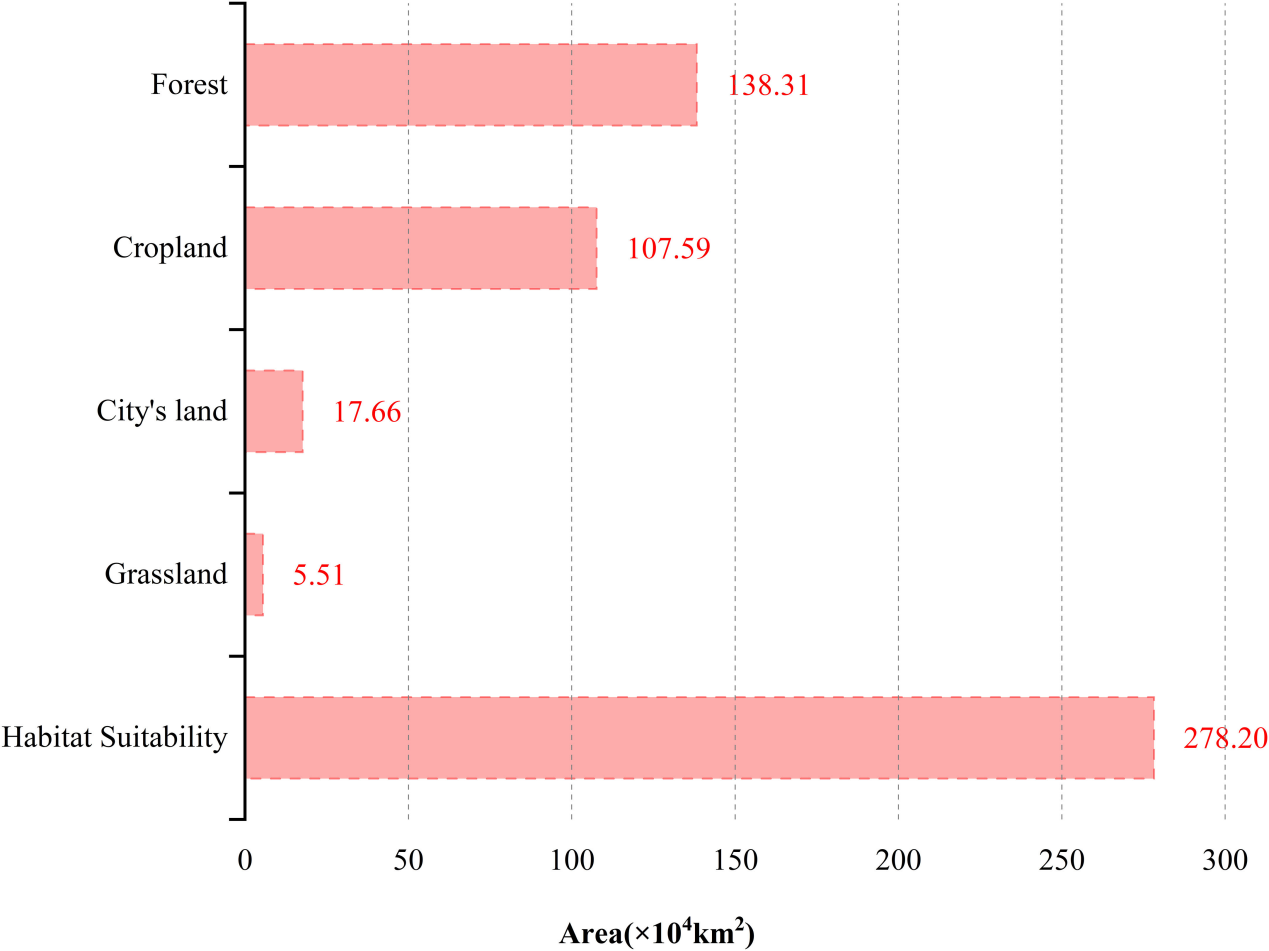
Area of forest, cropland, city’s land, and grassland at risk of invasion by *P. hysterophorus* in China.

**Figure 10:**
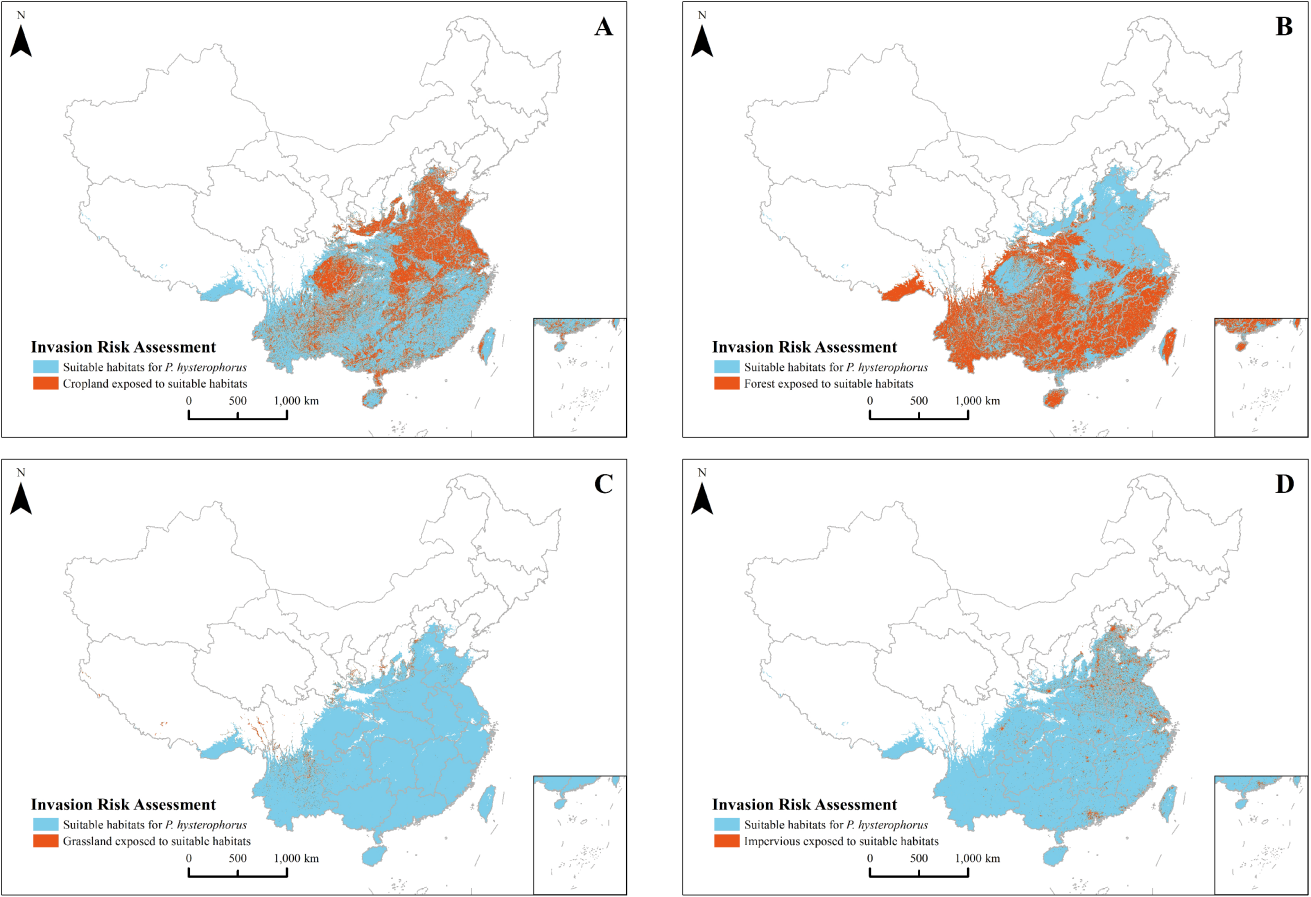
Distribution of cropland **(A)**, forest **(B)**, grassland **(C)**, and city’s lands **(D)** at risk of invasion by *P. hysterophorus* in China.

According to **Figure 9**, *P. hysterophorus* threatens various habitats including cities, croplands, forests, and grasslands. Forests, which constitute 50% of the potential invasion area, are the most at risk, followed by croplands at 39%, with cities and grasslands making up the remaining 11%. This underlines the importance of preparing for *P. hysterophorus* invasions, particularly in coastal agricultural lands, urban areas, and southwestern grasslands. Specifically, forests vulnerable to invasion span southeast Tibet, Henan, Zhejiang, southern Jiangsu, and most of Hainan, Yunnan, Guangxi, Guangdong, Fujian, Hunan, Sichuan, and Jiangxi, covering 139×10^4^ km² (**Figure 10B**). Croplands facing invasion risks are predominantly in the North China Plain—encompassing the Lower Liaohe Plain, Huangpan Plain, Huaibei Plain, and Haihe Plain—as well as the middle and lower reaches of the Yangtze River Plain. This includes the Jianghan Plain in Hubei, the Dongting Lake Plain in Hunan, the Poyang Lake Plain in Jiangxi, the Yangtze River Coastal Plain and the Chaohu Plain in Anhui, as well as the Yangtze River Delta region of Jiangsu, Zhejiang, and Shanghai. The Sichuan Basin, specifically the Chengdu Plain, is also affected, totaling an area at risk of 108×10^4^ km² (**Figure 10A**). Urban areas at risk include the coastal zones of Beijing, Shandong, Shanghai, Jiangsu, Zhejiang, Guangzhou, Fujian, and Taiwan, summing up to 18×10^4^ km² (**Figure 10D**). Grasslands at risk are mainly in the southwest, particularly parts of Yunnan and Tibet, amounting to 6×10^4^ km² (**Figure 10C**).

## 4 Discussion

As one of the world’s most problematic and pernicious invasive species, the invasive behavior of *P. hysterophorus* poses a serious threat to biodiversity, agricultural and forestry production, and human health (Bajwa et al., 2015), while the risk of its global invasion and spread is higher as a result of global climate and land-use change, and the increase in human activities such as international trade (Li et al., 2021; Xian et al., 2023). A number of scholars have already used SDMs to predict potential invasion areas of *P. hysterophorus* (Duque et al., 2022; Masum et al., 2022). In contrast to previous studies on the potential fitness zones of *P. hysterophorus*, our study not only predicts the potential distribution of *P. hysterophorus* under climate change scenarios, but also establishes a risk assessment for the species and evaluates the potential impacts on cities, croplands, forestlands, and grasslands in China (Kriticos et al., 2015). This in-depth approach distinguishes our study from previous ones that focused mainly on potential invasive areas of *P. hysterophorus*. Furthermore, our results are in line with Adhikari et al. (2023), highlighting the importance of environmental factors such as bio1, bio13 and bio3 on the distribution of *P. hysterophorus*. In addition, our study confirms the potential impact of climate change on species distribution, in line with the observed trend of species range expansion due to climate change (Chhogyel et al., 2021). However, the stage and extent of the invasion in China are still unclear (Liu et al., 2023). Therefore, a fundamental assessment of the risk of settlement and potential impacts of the species is crucial to prevent further spread (Egawa and Matsuhashi, 2022).

### 4.1 Key predictor variables affecting the potential distribution of *P. hysterophorus*

While most studies on SDMs typically choose only climatic variables for modeling and prediction, this study considered multiple factors, including climate, soil, topography, and vegetation. Based on the variable contribution results in this study, key drivers influencing the invasion of *P. hysterophorus* were identified to gain a deeper understanding of the environmental conditions conducive to the invasion. The prediction results showed that the main factors affecting the potential distribution pattern of *P. hysterophorus* were annual mean temperature (bio1), precipitation of the wettest month (bio13), isothermality (bio3), and temperature seasonality (bio4). In this study, we found that temperature and precipitation influence the growth and development of *P. hysterophorus*, with bio1 and bio13 being the most important variables affecting its potential distribution. Previous research indicates that, in the context of global climate change, many exotic invasive species can adapt more likely to climate change than native species. IAPs exhibit greater environmental tolerance and phenotypic plasticity to elevated temperatures, increased carbon dioxide concentrations, and changes in the environment (Molina-Montenegro and Naya, 2012; Nicotra et al., 2010). *Parthenium hysterophorus* has the capacity to thrive under extreme hot weather conditions (Kaur et al., 2017). The seed germination temperature for *P. hysterophorus* extends from 8°C to 35°C, accommodating a broad spectrum of temperatures (Tamado et al., 2002). Particularly during extreme weather conditions, the invasive weed modifies its reproductive growth, either delaying or accelerating it. This adaptive behaviour explains the species’ inherent adaptive potential, enabling widespread dispersion in non-native regions. Our findings revealed a bio1 range of 10.69 to 38.81°C, consistent with the characterised seed germination temperatures (Bajwa et al., 2018). Also, *P. hysterophorus* has a remarkable ability to germinate at low humidity levels and is more likely to survive in arid environments with excellent drought tolerance contrasted with native plants (Afzal et al., 2022). Consequently, changes in temperature and humidity conditions, including areas of increased temperature and decreased precipitation, may favor *P. hysterophorus* dispersal and growth because they are more tolerant and able to maximize suitability for new climatic changes. In particular, exotic plants of subtropical origin introduced to colder regions will increase their population size and number under climate change and spread over an extensive geographic range (Sheppard and Stanley, 2014).

Besides, among the many environmental variables, isothermality (bio3) and temperature seasonality (bio4) affect the potential distribution of *P. hysterophorus*. For bio3, the probability increased from 0.5 to 0.9, and the isothermality curve maintained an upward trend. The isothermality value increased from 27.32 to 90.80, reaching a point where the probability exceeds 0.9 and the response curve achieves stability. The continuous rise in isothermality indicates that diurnal temperature fluctuations are large relative to annual temperature fluctuations (i.e., higher isothermality), which suggests that *P. hysterophorus* is capable of sustained growth under long-term stressful conditions characterized by persistently decreasing diurnal and annual temperatures, consistent with the previous study findings (Jin et al., 2022; Qi et al., 2022; Shrestha et al., 2024). For bio4, the range of its curve is less variable compared to the previous variables, suggesting that its temperature seasonality is less limiting to the potential distribution probability of *P. hysterophorus* and that temperature seasonality is not significant. It is now well documented that under the general warming trend, the minimum temperature rises about twice as fast as the maximum temperature, the diurnal temperature range gradually decreases, and isothermality and temperature seasonality are not distinct, thus leading to warm and humid climates in the tropics and sub-tropics (Chemke and Yuval, 2023; Frierson et al., 2013; Walther et al., 2002). In such environments, *P. hysterophorus* as an exotic species are better able to colonize and expand, eventually becoming invasive (Pys̆ek et al., 2002). Simultaneously, warmer temperatures will result in shorter winters and earlier spring phenology. It will significantly shorten the dormancy of *P. hysterophorus* seeds, enabling them to germinate and establish as plants in early summer (Karlsson et al., 2008; Williams and Groves, 1980). Therefore, climate change is a crucial driver of invasive plant behavior and the invasiveness of plants may be intensified by their invasion capabilities (Parmesan and Hanley, 2015). Despite the inherent uncertainty of climate change affecting the spread of *P. hysterophorus* in China. Based on the potential distribution of *P. hysterophorus*, a comprehensive survey of IAPs can be undertaken at high-risk cities, which can be controlled within a certain range to prevent it from spreading further and affecting other cities. In cities with a medium risk of occurrence, emphasis should be placed on monitoring and observation, with the execution of timely cleanup and disinfestation measures to minimize future management costs. For cities at low risk, educational and publicity initiatives will be conducted, and preventative and control measures implemented in advance.

### 4.2 Invasive historical reconstruction

The reconstruction of *P. hysterophorus* dispersal routes have unveiled invasion patterns across China. First, the observed dispersal mirrored stochastic simulations, highlighting a structured pattern in the dispersal dynamics of *P. hysterophorus*. Second, *P. hysterophorus* first invaded and spread to areas highly suitable, with climatic conditions akin to its native range. Following the initial dispersal phase, where the species proliferated across southern China, its presence in areas with dissimilar climatic conditions grew increasingly prevalent. *Parthenium hysterophorus* has gradually enhanced its dispersal (Phillips et al., 2006a), growth (Burton et al., 2010), and adaptive capabilities, due to a simple evolutionary process stemming from reproduction among the most effective dispersers at the colonization frontier (Colautti and Barrett, 2013; Shine et al., 2011). The total observed dispersal route length is higher than the minimum possible length in China. This implies a frequent occurrence of long-distance dispersal, underscoring the significant role of trade and transportation of goods in *P. hysterophorus*’ spread across China. For instance, *P. hysterophorus* was reported to have migrated from Vietnam to Yunnan Province in southern China between 1924 (Tang et al., 2009) and 1926 (Gao et al., 2013), probably aided by road traffic (Nguyen, 2011). Furthermore, in 2004, a separate population was introduced into Shandong Province in northern China. Speculation suggests that this population was likely introduced via a major port in Lianyungang, Jiangsu Province, situated 150 km from Shandong Province (Shou et al., 2014).

### 4.3 The potential invasive distribution of *P. hysterophorus* under current climatic

We observed that the invasive characteristics of *P. hysterophorus* display a condition trend in China, with more prevalence in the southeast and less in the northwest, mainly concentrated in coastal cities, while the intensity and area of suitable habitat gradually decrease from the coast to inland. This is consistent with the results of our response curves, and it is possible that *P. hysterophorus* is gradually spreading to cooler and drier environments. *Parthenium hysterophorus* is native to the American tropics and prefers sunny and moist environments (Kohli et al., 2006). This growth characteristic makes them prefer to grow in southeastern China, thus showing a trend of increasing growth intensity and suitable area in this region. It is the result of the subtropical monsoon climate in southeastern China, characterized by hot summers and warm winters, four distinct seasons, simultaneous rain and heat, and developed monsoon winds, creating an ecological environment with superior thermal and hydrological conditions. Water serving as a crucial dispersal route for *P. hysterophorus* seeds, plays a key role in shaping the dispersal of *P. hysterophorus*. In the monsoon or rainy seasons, *P. hysterophorus* seeds are able to spread through river systems. Even during dry periods, they can come into contact with the soil, thereby continuously increasing the size of their soil seed bank (Li and Qiang, 2009; Mao et al., 2019, 2022; Zhang et al., 2019). In surveys of dry savanna reserves, *P. hysterophorus* also often spreads into open dry savannas with seasonal streams (Pyšek et al., 2020). It confirms our research that coastal areas are more vulnerable to invasion by *P. hysterophorus* than inland dry areas. Therefore, in the future, measures such as regular removal of plants from river channels, particularly after floods to minimize their spread, consistent monitoring of river and irrigation channel systems, and the early identification of areas that may be affected by the spread of invasive species seeds.

### 4.4 The potential invasion dynamics under future climate

Global climate change, which often comes with environmental challenges like increasing temperatures, a rise in extreme weather events, and increasing soil salinity, can cause harm to exist ecosystems and pose a threat to the growth of native plants (Di Capua and Rahmstorf, 2023; Sheppard et al., 2012; Thuiller et al., 2007). Nevertheless, *P. hysterophorus* is becoming increasingly resilient to its environment, displaying a broad geographic range, wide climate tolerance, and resistance (Bajwa et al., 2019b; Cowie et al., 2020). For example, tolerance to temperature stress is critical for plant germination and survival of seedlings (Hou et al., 2014). The survival of *P. hysterophorus* in these very hot and dry areas suggests that could potentially survive under more extreme high temperatures conditions than previously thought (Shabbir et al., 2023). It is consistent with our findings that under the influence of future climate change, there is a general upward trend in the mid-to high-performing area of *P. hysterophorus*, with the range gradually expanding from low to mid- to high-latitude districts. Not only that, but warming will lead to a rise in sea level, which will trigger the salinization of coastal soils, although salinity had a negative effect on different growth variables elevated CO_2_ improved *P. hysterophorus*’s growth and phenolics content regardless of the salt stress regime. It may benefit from its resistance to invade these areas (Saravanane et al., 2023). Coastal cities in China, except for Liaoning Province, have proven to be within the invasive range of the *P. hysterophorus*. Hence, it is essential to intensify monitoring and preventive efforts in new potential distribution areas at higher latitudes to minimize the risk of spreading invasion. Furthermore, enhancing surveillance of sea surfaces in coastal regions and implementing protective measures for vulnerable ecosystems is crucial. Restoring and safeguarding wetlands and mangrove forests will not only maximize the prevention of *P. hysterophorus* invasion but also slow soil salinization. Collectively, these measures will safeguard local ecosystems against the detrimental impacts of *P. hysterophorus*.

### 4.5 Invasion risk associated with cities, croplands, forestlands, and grasslands

In this study, we paid particular attention to the possible invasion risk of *P. hysterophorus* to the cities, croplands, forests, and grasslands of China, where its invasion could pose a severe health threat and damage to the environment, ecosystem functions, crop yields, crop production, habitat scarcity, livestock performance, land degradation, and acute health hazards complications in cellular (Batish et al., 2007). According to the latest records, in 2019, an expert performed an ecological survey of invasive alien species at the Chengdu Customs site in Sichuan, China, uncovering a *P. hysterophorus* plant population. This discovery shows that *P. hysterophorus* is still expanding its invasive range in China. The invasion risk assessment indicates that China should be on alert for the invasion of *P. hysterophorus* in cities, the North China Plain, the Middle and Lower Yangtze River Plain and the Chengdu Plain farmlands, the woodlands in the southern region, and the grasslands in the southwestern region (**Figure 9**). In addition, woodlands have the highest area at risk.

We found that most of the forest land in southern China is within the invasion range of *P. hysterophorus*, which is a challenge to the forest ecosystem (**Figure 10B**). In particular, most of the forests in the south of China are subject to road and urbanization development, with high fragmentation, and the forest ecosystems have become fragile. Moreover, as an aggressive invader with exuberant growth habits, *P. hysterophorus* disrupts the structure of natural ecosystems and displaces numerous native plant species from those ecosystems (Adkins and Shabbir, 2014; Gnanavel, 2013; Shabbir et al., 2021), thereby breaking the balance of the forest ecosystem. This invasive weed has a negative impact on forest ecosystems and has become a major threat to forest reserves around the world (Shang et al., 2022; Strathie et al., 2011). Apart from the lower plant diversity and unfavorable growth of local plants in the areas invaded by *P. hysterophorus*, there have been alterations in soil chemical properties, characterized by decreased pH values, phenolic compounds, organic matter content, and concentrations of nitrogen, phosphorus, and potassium (Boja et al., 2022; Kaur et al., 2019).

The safety of cropland should also be a top priority. Healthy arable land is vital for ecological, economic, and socio-economic development. This study uncovered that *P. hysterophorus* invasion encompasses a substantial portion of land used for grain production, encompassing nine major grain-producing regions in China (**Figure 10A**), namely Henan, Shandong, Sichuan, Jiangsu, Hebei, Anhui, Hunan, Hubei, and Jiangxi. These regions experience consistently high temperatures, minimal annual temperature fluctuations, well-established water systems, fertile soils, and a suitable climate for grain cultivation. However, research shows that *P. hysterophorus* inhibits the growth of maize, rice, and forage grass. With its density increases, it inhibits crop growth, reduces crop yields, and increases competition with crops (Bajwa et al., 2019a; Khan et al., 2013; Safdar et al., 2015). As reported by Pratt et al. (2017) annual economic losses to maize crops caused by *P. hysterophorus* ranged from 46.6 to 71.4 million dollars in Ethiopia, 3.8 to 7.7 million dollars in Kenya, 0.3 to 1.0 million dollars in Tanzania, and 0.7 to 1.8 million dollars in Uganda. Additionally, Kumar et al. (2016) discovered that *P. hysterophorus* can carry Tomato yellow leaf curl virus (TYLCV), whose infection induces leaf curl symptoms in *P. hysterophorus* and tomatoes. It shows that *P. hysterophorus* can serve as a host for certain viruses, thereby increasing the risk of spreading tomato leaf curl disease to some extent. In our research, we have also observed that *P. hysterophorus* is widely distributed in economically developed regions and areas with relatively high population density, posing a threat to human life and health due to its invasion (**Figure 10D**). It is worth noting that *P. hysterophorus* is often found in open fields and along roadsides in the field. However, in most cases, it gradually spreads to nearby cities areas under the influence of human activities, disrupting native plant ecosystems (Appalasamy et al., 2020; Potgieter et al., 2019). The invasion process is driven by a combination of environmental and anthropogenic factors, with environmental factors determining how many species can survive in a new location, and anthropogenic factors affecting the timing, quantity, and route of species introduction to the site (Chen et al., 2021). In our study, we also found that *P. hysterophorus* is abundantly distributed in economically developed areas and areas with relatively high concentrations of population density which often results in conditions such as eczema, skin inflammation, and respiratory diseases that endanger human safety and health (Akham et al., 2023). The results that the grasslands invaded by the *P. hysterophorus* predominantly occupy low-elevation areas in southwestern China, notably within Yunnan and Sichuan Provinces, which offer suitable conditions for cattle and sheep farming (**Figure 10C**). However, excessive grazing has exacerbated the invasion of *P. hysterophorus*, resulting in higher plant density, larger plants, and increased seed production. This over-invasion is also evident in alterations to surface plant communities, leading to a notable decline in species richness, evenness, and diversity (Shabbir et al., 2019). Furthermore, the pollen carried by *P. hysterophorus* itself can induce dermatitis in animals. Excessive consumption by animals can lead to mouth ulcers and excessive salivation, resulting in poisoning (Kaur et al., 2014).

Therefore, enhancing control of existing invasive alien species is imperative. Through monitoring, hazard surveys, hazard assessments, and risk analyses, it is critical to identify sources and mitigate further proliferation and spread. Simultaneously, prioritizing international and domestic quarantine is essential to prevent the introduction of new species and to curtail hybridization and gene flow between populations in different regions (Tang et al., 2009; Fan et al., 2018).

### 4.6 Limitations of this study

First, this study only used the MaxEnt model to simulate and predict the distribution of *P. hysterophorus*. Previous studies have found that the prediction results of an ensemble model will outperform a poorly performing single model and underperform a better performing single model (Zhu and Peterson, 2017). However, this study indicated that the MaxEnt model could meet the demand in simulating the distribution of *P. hysterophorus* with a high accuracy. Secondly only environmental variables were considered in our study. We ignore the effects of human activities on the dispersal of *P. hysterophorus*, such as livestock and motorized vehicles, which can spread large quantities of seeds of many plant species over great distances (Auffret et al., 2014). The relationship of these dispersal vectors to the physical environment may influence the movement of the seeds they spread. Third, the uncertainty in predicting the potential invasive potential distribution of *P. hysterophorus* arises from the multifaceted nature of future climate change and the variability among different global circulation models (GCMs). Utilizing integrated simulations that combine multiple GCMs could substantially enhance the certainty and accuracy of such predictions (Puchałka et al., 2023a, 2023). Forth, the ecological niche requirements of species are conservative, and we ignore the potential adaptive capacity of species to new environments. There may be differences in climatic ecological niches between the place of origin and the place of invasion, resulting in the phenomenon of “ecological drift” (Manzoor et al., 2020; Xian et al., 2023). Last, although we realize that in reality, NDVI is likely to change with climate change, we maintain the assumption of constant NDVI in the model based on currently available data and practical considerations for model construction. In fact, land-cover conversion is likely to occur in the future, which will result in altered vegetation physiology (Defries et al., 2002). Future research on the potential invasion potential distribution of *P. hysterophorus* should adopt this integrative approach. Although there are some limitations in our study, we still predict the invasion dynamics of *P. hysterophorus* in China under climate change. And the results of this study were the first step of the macro-planning, and still had important guiding significance for the management of *P. hysterophorus*.

## 5 Conclusion

In this paper, we reconstructed the dispersal routes of *P. hysterophorus* in China and used the optimal MaxEnt model to predict the potential distribution of *P. hysterophorus*, a significant invasive alien weed in China, based on the occurrence of the screened species and related environmental variables. Our analysis of dispersal routes revealed structured dispersal dynamics and suggested a human influence on the spreading of *P. hysterophorus* across China. Furthermore, the environment variables, especially mean annual temperature (bio1) and the wettest monthly precipitation (bio13), serve as the primary factors influencing the potential distribution of *P. hysterophorus*. Moreover, under the current climate, southeastern China emerges as the principal potential invasion area for *P. hysterophorus*. With the onset of climate change, its potential distribution range will extend to higher latitudes. Continuous monitoring and enhanced management of forests and agricultural lands should be prioritized, as these areas are particularly susceptible to the invasive impacts of *P. hysterophorus*. Proactive measures are essential to prevent its further spread and invasion. The results of our study provide valuable insights into the potential distribution patterns and underlying factors driving *P. hysterophorus* invasion. This information lays the groundwork for the development of effective control measures in the future and serves as a valuable scientific reference for the prevention and management of biological invasions.

## Data Availability

The original contributions presented in the study are included in the article/**Supplementary Material**. Further inquiries can be directed to the corresponding author.
