# Supplementary material for: Reconstructing the biological invasion of noxious invasive weed Parthenium hysterophorus and invasion risk assessment in China
Source: Front Plant Sci. 2024 Sep 19;15:1430576. doi: 10.3389/fpls.2024.1430576 (PMC11446801; doi:10.3389/fpls.2024.1430576)
Supplement: Supplementary file 1 [file DataSheet1.docx]

Supplementary Material

# Supplementary Figures and Tables

## Supplementary Figures


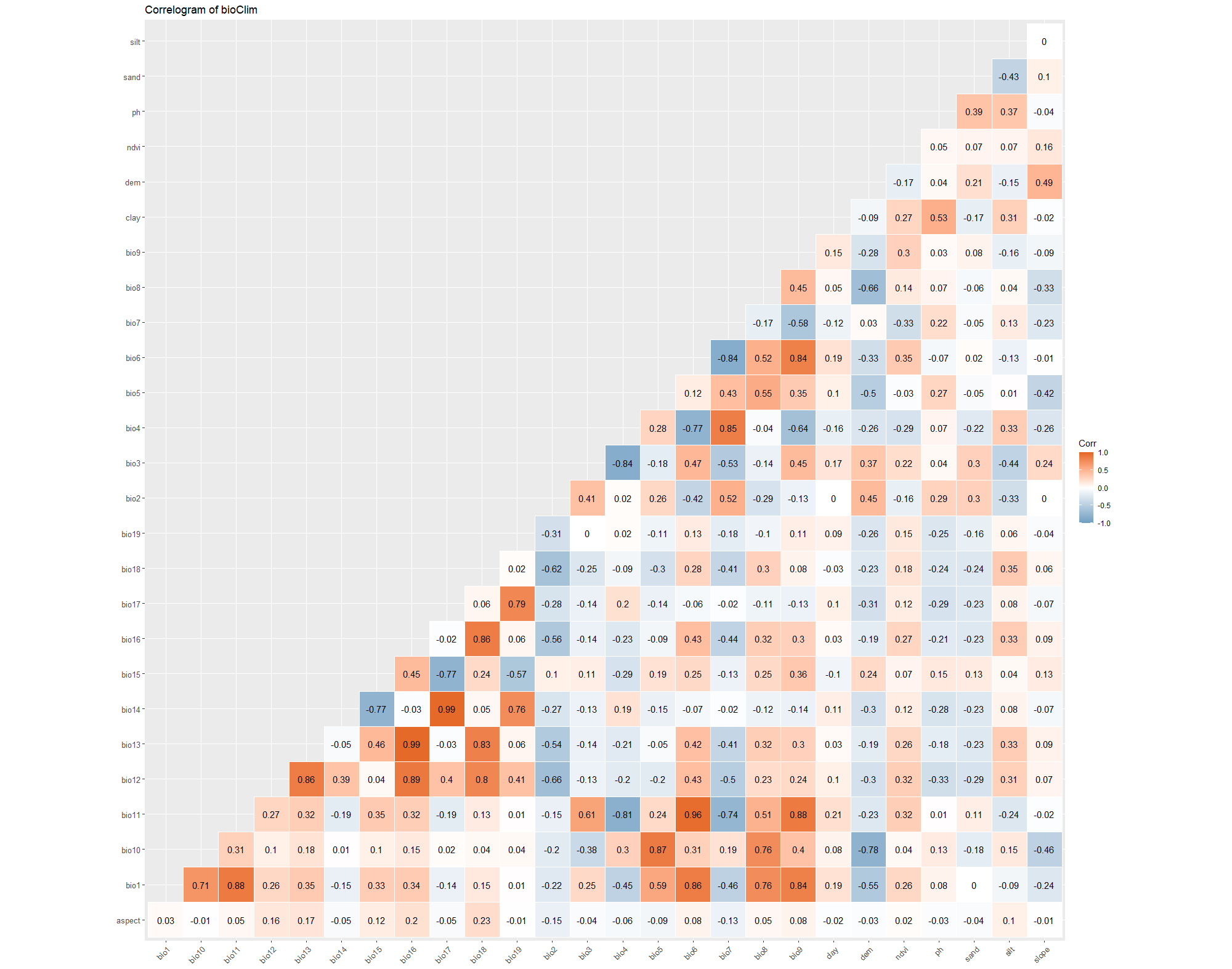


**Supplementary Figure 1.** The correlation among all used environmental variables.

## Supplementary Tables

**Supplementary Tables 1.** Sample Sections Survey

| **Number** | **Species** | **Longitude** | **Latitude** | **Year** | **City** |
| --- | --- | --- | --- | --- | --- |
| 1 | *Parthenium hysterophorus* | 113.7068 | 23.22878 | 2022 | Guangzhou |
| 2 | *Parthenium hysterophorus* | 112.319 | 22.16576 | 2022 | Jiangmen |
| 3 | *Parthenium hysterophorus* | 110.869 | 22.20766 | 2022 | Maoming |
| 4 | *Parthenium hysterophorus* | 111.1552 | 21.47395 | 2022 | Maoming |
| 5 | *Parthenium hysterophorus* | 115.7686 | 24.079 | 2022 | Meizhou |
| 6 | *Parthenium hysterophorus* | 110.8418 | 21.52389 | 2023 | Zhanjiang |
| 7 | *Parthenium hysterophorus* | 110.1478 | 20.86488 | 2023 | Zhanjiang |
| 8 | *Parthenium hysterophorus* | 110.2652 | 20.77129 | 2023 | Zhanjiang |
| 9 | *Parthenium hysterophorus* | 110.1288 | 20.79839 | 2023 | Zhanjiang |
| 10 | *Parthenium hysterophorus* | 110.1242 | 20.99101 | 2023 | Zhanjiang |

Importance values for herbs were calculated in 1 x 1m squares. Determine the dominant species in the sample based on the top 10% of the importance values.

**Supplementary Tables 2.** Environment Variables Data Sources

| Variables | Abbreviations | Data sources |
| --- | --- | --- |
| 19 bioclimatic variables | bio | https://www.worldclim.org/ |
| Terrain | dem | https://cmr.earthdata.nasa.gov/search/concepts/C1546314043-LPDAAC_ECS.html |
|  | slope | Analyzed based on dem |
|  | aspect | Analyzed based on dem |
| Environment | ndvi | https://data.nasa.gov/dataset/MODIS-Terra-Vegetation-Indices-16-Day-L3-Global-50/diay-vffa/data |
| Soil quality | sand | https://www.isric.org/ |
|  | clay |  |
|  | silt |  |
|  | pH |  |

**Supplementary Tables 3.** Environment Variables Data Sources

| **Variable** | **Percent contribution** | **Permutation importance** | **Variable** | **Percent contribution** | **Permutation importance** |
| --- | --- | --- | --- | --- | --- |
| bio1 | 37.5 | 12.3 | bio14 | 0.3 | 2.6 |
| bio11 | 23.3 | 8.4 | slope | 0.3 | 1.2 |
| bio18 | 9.5 | 5 | dem | 0.3 | 1.2 |
| bio13 | 5.4 | 0.3 | clay | 0.3 | 5.4 |
| bio4 | 4.5 | 2.9 | bio12 | 0.2 | 0.9 |
| bio17 | 3.5 | 7.7 | silt | 0.1 | 1 |
| bio19 | 3.4 | 3.4 | bio10 | 0.1 | 0.5 |
| bio8 | 2.6 | 8.1 | bio15 | 0.1 | 0.8 |
| bio3 | 2.1 | 12.1 | bio2 | 0.1 | 0.3 |
| bio16 | 2 | 5.8 | bio9 | 0 | 1 |
| sand | 1.8 | 1.4 | bio5 | 0 | 0.1 |
| ndvi | 1.4 | 5.2 | bio7 | 0 | 0.1 |
| bio6 | 0.9 | 6.9 | aspect | 0 | 0.1 |
| ph | 0.5 | 5.5 |  |  |  |

**Supplementary Tables 4.** Environment Variables Data Sources

| **Variable** | **Percent contribution** | **Permutation importance** |
| --- | --- | --- |
| bio1 | 36.4 | 40.6 |
| bio13 | 34.6 | 11.7 |
| bio3 | 11.7 | 11.3 |
| bio4 | 6 | 10.9 |
| bio19 | 6 | 6 |
| ndvi | 2.3 | 2 |
| bio8 | 1.9 | 15.3 |
| sand | 1.2 | 2.2 |
